# The Impact of Patient-Generated Health Data From Mobile Health Technologies on Health Care Management and Clinical Decision-Making: Narrative Scoping Review

**DOI:** 10.2196/77359

**Published:** 2025-12-19

**Authors:** Ava Keeling, John Downey, Matthew Halkes, Yinghui Wei

**Affiliations:** 1 Centre for Mathematical Sciences, School of Engineering, Computing and Mathematics, University of Plymouth, Kirkby Place, Drake Circus, Plymouth, PL4 8AA, United Kingdom; 2 Nuffield Department of Population Health, University of Oxford, Oxford, United Kingdom; 3 Leverhulme Centre for Demographic Science, University of Oxford, Oxford, United Kingdom; 4 Centre for Health Technology, School of Nursing and Midwifery, University of Plymouth, Plymouth, United Kingdom; 5 Anaesthetics, Torbay and South Devon NHS Foundation Trust, Torbay, United Kingdom; 6 Exeter Medical School, Faculty of Health and Life Sciences, University of Exeter, South Cloisters, St Luke’s Campus, College Road, Exeter, EX1 2LU, United Kingdom, 0441392724929; 7 NIHR Applied Research Collaboration South West Peninsula, Exeter, United Kingdom

**Keywords:** clinical decision-making, health care management, long-term conditions, mobile health, multimorbidity, patient-generated health data, service design, Preferred Reporting Items for Systematic Reviews and Meta-Analyses, PRISMA

## Abstract

**Background:**

Long-term health conditions and multimorbidity are increasing globally, placing an unsustainable pressure on health care systems. Mobile health (mHealth) technologies enable the collection of patient-generated health data outside clinical settings, offering the potential to support personalized care and inform clinical decision-making. However, the ways in which mHealth patient data are being used in clinical practice remain unclear.

**Objective:**

This study aimed to map and synthesize the existing literature on how patient-generated mHealth data are reportedly being used and influencing clinical decision-making for adults with long-term conditions in an outpatient care setting.

**Methods:**

A narrative scoping review was conducted on studies published between 2014 and 2025. Studies were eligible for inclusion if they were in English, had data on the use of patient-generated mHealth data, went beyond feasibility testing, and had reference to clinician behavior or patient interactions. Gray literature was not used to maintain a focus on peer-reviewed and published evidence. Studies involving pediatric or adolescent populations were excluded. Searches were conducted across the following databases between 2014 and 2025: Embase, MEDLINE, Knowledge and Library Hub, British Nursing Index, and ProQuest Health Research Premium Collection. Data were charted systematically and synthesized narratively. Key data included study characteristics, mHealth use, data types and visualizations, patient demographics, and the ways the data informed clinical decision-making.

**Results:**

A total of 16 studies met the inclusion requirements, which were primarily high-income countries focusing on rheumatoid arthritis and diabetes. The studies reported on how mHealth data were integrated into workflows, influenced health care decisions, and shaped patient-provider interactions. mHealth patient data were found to support patient-centered care and facilitate proactive holistic care, though in some instances, the data were shown to reinforce medical agendas removing agency from patients. There is also a gap between the intended use of the data and their implementation in clinical practice. The reported barriers included professional skepticism, integration challenges, and concerns about data accuracy. Evidence was focused on feasibility rather than long-term outcomes, with limited evidence on the impacts of mHealth.

**Conclusions:**

Patient-generated health data have the potential to enhance clinical decision-making and person-centered practices. However, integration into routine practice is hindered by technological challenges, professional hesitancy, and a lack of standardization. Future research should prioritize supporting integration, improve data presentation, and evaluate the long-term effects on clinical workflows. Addressing these barriers and establishing clear policy frameworks will be crucial for realizing the potential of mHealth in health care delivery.

## Introduction

Long-term health conditions (LTHCs), which include chronic diseases such as diabetes, cardiovascular disease, and rheumatoid arthritis, continue to represent a large portion of the global burden of disease [1]. The prevalence of LTHCs and multimorbidity, characterized by the presence of 2 or more LTHCs in an individual, is increasing rapidly due to factors such as lifestyle behaviors [2] and the aging population [3], magnifying the pressures on health systems. LTHCs negatively impact life expectancy, quality of life, and mental health [4]. Additionally, multimorbidity is also linked to poor clinical outcomes, in part due to the substantial amount of self-management required when living with multiple health conditions [5].

The experience of living with LTHCs and multimorbidity is frequently characterized by fragmented health care services with limited communication between different health care providers [6]. The increasing use and availability of mobile health (mHealth) technologies have the potential to transform the landscape of health care delivery, particularly in the management of LTHCs and multimorbidity [7] while also helping to address the growing imbalance between health care demand and system capacity. Patient-generated health data (PGHD), including patient-reported outcome measures, patient-reported experience measures, and physiological data, are now routinely collected outside of clinical settings through mHealth apps; however, the utilization of these data in the service delivery is yet to be widely explored [8]. For consistency, PGHD are used throughout this review to refer to patient-generated data collected via mHealth technologies, unless otherwise specified by the original authors. Nascent literature has shown that mHealth has the ability to improve the quality of patient care, increase patient-provider communication, and achieve better health outcomes throughout a patient’s disease journey [9].

While the potential of PGHD to enhance clinical decision-making, health care management, and service design has been widely acknowledged, the extent and nature of the actual influence remain unclear [8]. In this review, health care management refers to the organization, coordination, and delivery of health services, including resource allocation, care pathway design, triage processes, and population health management strategies. The integration of PGHD into outpatient care pathways promises to inform triage, support risk prediction, and guide personalized treatment strategies [10]. Furthermore, at a system level, such data may support population health management and service transformation. Nonetheless, questions remain regarding how PGHD are operationalized in practice, how PGHD interact with existing clinical hierarchies, and whether PGHD deliver on their promises. Although the authors acknowledge there are wider drivers of how PGHD may alter health care management, including fiscal benefits for primary care, diverse insurance models or incentives, and resourcing issues concerning the costs of mHealth, the current review was preoccupied with charting the role of PGHD in influencing clinician delivery alone. The authors do, however, recognize the wider determinants of how PGHD may impact engagement and outcomes.

While there is a growing interest in the use of PGHD in the management of LTHCs, the literature examining its influence on clinical decision-making remains sparse and fragmented. Many studies focus on the role of PGHD in individual health conditions or patient outcomes [11], but few have comprehensively examined its broader implications for health care delivery. Given the limited number of studies and the broad, varied nature of the existing evidence, a systematic review would be too narrow to adequately capture the full scope of this field. Instead, a scoping review is more relevant, as it allows for the exploration of a wider range of perspectives and status quo of the field.

The current narrative scoping review explored how PGHD are influencing clinical decision-making and delivery for adults with long-term conditions in outpatient settings. By mapping the current literature, this review highlights key mechanisms through which PGHD contributes to care transformation, the tensions that emerge in their application, and the gaps that warrant further longitudinal evaluative research.

## Methods

### Review Design

A narrative scoping review was undertaken in line with recommendations from Pollock et al [12], Arksey and O’Malley [13], and the PRISMA-ScR (Preferred Reporting Items for Systematic Reviews and Meta-Analyses extension for Scoping Reviews) checklist [14], and a protocol was not registered a priori. A narrative analysis approach was chosen for this review due to the limited and heterogeneous nature of the existing literature and phenomena of interest. In practice, the 5 stages outlined by Arksey and O’Malley were followed: identifying the research question, identifying relevant studies, selecting studies, charting the data, and collating or summarizing results. The final stage was adapted to use narrative qualitative synthesis to integrate the findings across diverse study types.

### Search Strategy

A comprehensive database search was performed with the support of a National Health Service librarian to identify relevant studies on December 8, 2023. The search aimed to retrieve papers exploring the impact of PGHD collected via mobile apps on clinical decision-making and service design. The search contained both natural language and controlled vocabulary terms and was performed across the following databases: Embase, MEDLINE, Knowledge and Library Hub, British Nursing Index, and ProQuest Health Research Premium Collection. Key search terms included combinations of “mHealth,” “patient-generated health data,” “long-term conditions,” “multimorbidity,” and “clinical decision-making.” Terms relating to “long-term conditions” and “multimorbidity” were explicitly included to ensure alignment with the review focus. The full search strategy, including specific Medical Subject Headings (MeSH) and keywords used for each database, is provided as a Supplementary File in Multimedia Appendix 1. Once the search was completed, the librarian removed duplicate records and provided an RIS file containing the final set of results. An additional 98 papers were also identified separately by the authors in a PubMed database search, 26 of which met the inclusion criteria. The search was carried out again on September 17, 2025, to capture any developments in the field since 2023. The papers were imported into Rayyan, and the removal of deduplicates was undertaken.

### Inclusion and Exclusion Criteria

When reviewing papers, the inclusion and exclusion criteria were discussed among the authors. There were no limits on paper type to capture the broadest range of relevant literature. Papers were included if they were published in English and provided sufficient details on how PGHD had been used to inform clinical decision-making. The papers that mentioned this in the title or abstract but did not meaningfully explore its application were excluded. Table 1 provides the detailed criteria that informed paper selection. Gray literature was not used to maintain a focus on peer-reviewed and published evidence. Adults were chosen as LTHCs disproportionately affect older adults [15].

**Table 1.** Criteria used to screen studies for the narrative scoping review on the impact of PGHD^a^ from mHealth^b^ technologies on health care management and clinical decision-making.

Criterion	Inclusion	Exclusion
Population	Adults with long-term conditions or multimorbidity	Pediatric or adolescent populations
Setting	Outpatient management	Virtual wards or acute settings
Intervention	Use of mHealth platforms or apps	Non-mHealth interventions
Data type	Studies involving PGHD	eHealth and other ICT^c^ research
Purpose of PGHD use	Pathway redesign or transformation; supported clinical decision-making or triage of care; population health management	—^d^
Language	English	Non-English
Publication type	Peer-reviewed journal papers	Gray literature

a PGHD: patient-generated health data.

b mHealth: mobile health.

c ICT: information and communication technology.

d Not available.

### Screening Process

Title and abstract screening were performed first by 3 reviewers (AK, JD, and MH) using Rayyan as a platform to track the process. Papers were organized alphabetically by author name, and each reviewer was allocated one-third of the papers. Authors independently screened the allocated papers as well as a second group that was allocated to one of the other reviewers, ensuring that each paper was screened by at least 2 reviewers. The reviewers then met to discuss any conflicts, ensuring that there was a consensus reached for inclusion decisions. Papers that passed the title and abstract screening were retrieved for full-text review. The division of work was the same. A PRISMA flow diagram was developed to help visualize the flow of the papers throughout the screening phases.

### Data Extraction

Data were extracted systematically into a structured data extraction spreadsheet by 1 reviewer (AK) (Multimedia Appendix 2). The process began with the recording of essential study details and key information about each paper. Any raw text viewed as pertaining to the research question was also noted and subsequently taken through to analysis. The reviewer also noted the ways in which mHealth had been used throughout the study. This was followed by identifying specific data types and any visualizations that had been used for the clinicians throughout the paper and identifying the cohort demographics.

The authors extracted relevant extracts verbatim, and each quote was provided a detailed qualitative code or memo for further analysis. Extracted data were coded according to whether PGHD informed pathway redesign or transformation, supported decision-making or triage of care, or contributed to population health management, ensuring coherence between the inclusion criteria and the synthesis.

### Data Synthesis

A qualitative analysis was performed by 2 reviewers (AK and JD) to identify key patterns in how PGHD collected via mHealth influenced clinical decision-making and delivery. This was initially performed by 1 reviewer, ensuring a structured approach to data synthesis. To enhance rigor and reliability, the identified themes were then cross-checked by a second reviewer, reducing the risk of bias and ensuring consistency in theme identification.

The analysis followed the principles of qualitative content analysis and constant comparison, allowing for the systematic identification and refinement of key themes across the included papers [16]. This iterative process enabled the grouping of findings into broader conceptual categories that reflected how PGHD were used in health care management and decision-making.

## Results

### Study Selection

**Figure 1.**
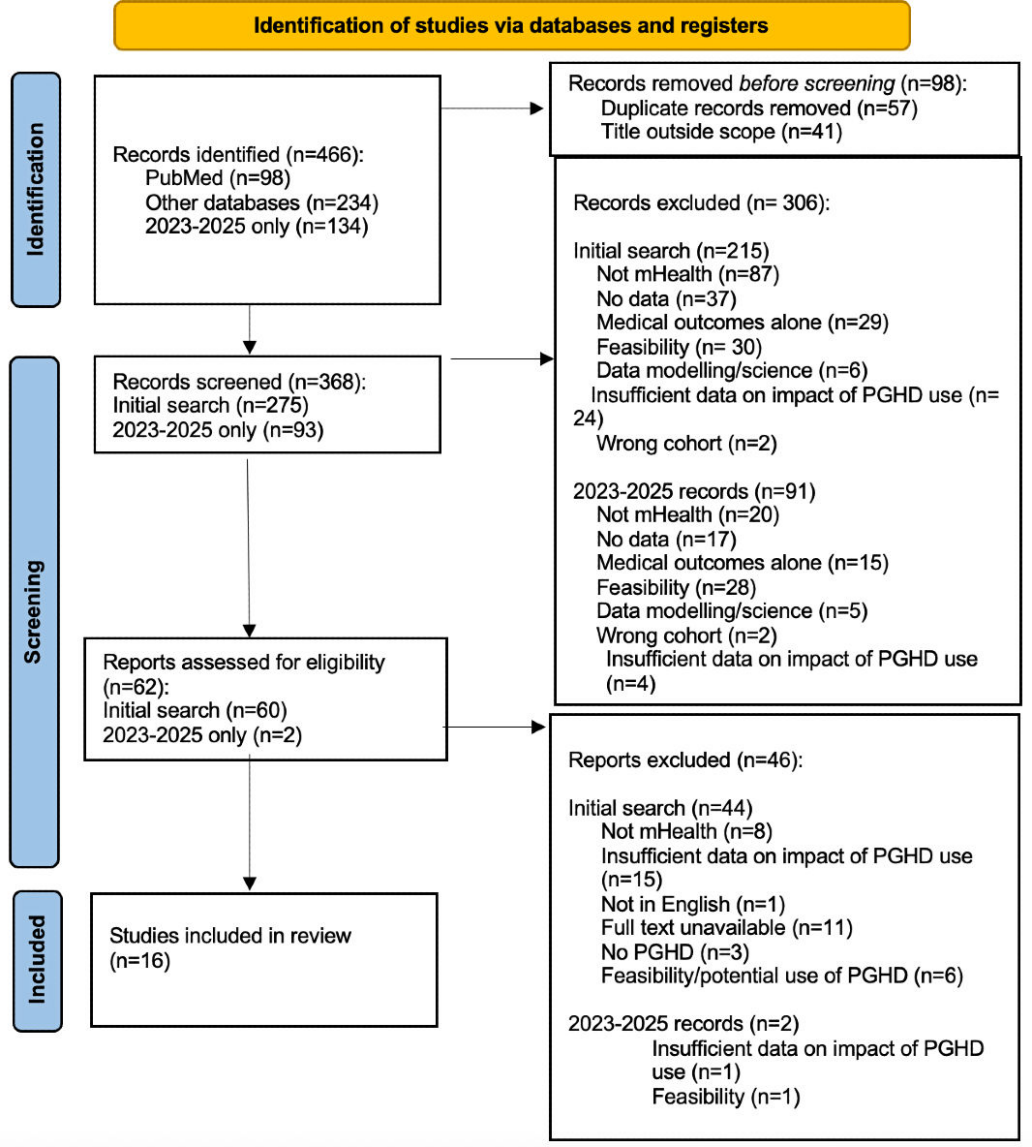
PRISMA (Preferred Reporting Items for Systematic Reviews and Meta-Analyses extension for Scoping Reviews) flow diagram illustrating the identification, screening, and inclusion of the studies in the narrative scoping review of the impact of patient-generated health data (PGHD) from mobile health (mHealth) technologies on health care management and clinical decision-making.

The database search yielded a total of 332 publications with 57 duplicates, meaning 275 publications remained. Title and abstract screening excluded 215 records, as many of the papers were not related to mHealth, were protocol or opinion pieces, captured medical outcomes or no clinician impact, or tested the feasibility of mHealth as a viable option within this setting alone, leaving 60 texts for full-text review (Figure 1). A further 44 studies were excluded due to reasons including insufficient data on the impact of PGHD use (n=15), not using mHealth interventions (n=8), having no English or full-text version available (n=12), and only focusing on feasibility or potential use of PGHD (n=6). This resulted in 16 studies included in this review, which are summarized in Table 2 (Figure 1). The subsequent search from 2023 to 2025 yielded 134 citations, of which 93 were within scope. Title and abstract screening indicated that most papers had the same issues as the original search, including feasibility or satisfaction testing within mHealth, protocols papers, and reporting clinical and service use outcomes alone. The literature has progressed, and more mHealth interventions, predictive modeling, PDHG adherence, and patient-initiated follow-up papers were noted. Around 2 papers progressed to full-text screening, but neither had sufficient information concerning how PGHD interacted with clinical behavior or delivery.

**Table 2.** Summary of the studies included in the narrative scoping review examining the impact of PGHD^a^ from mobile health (mHealth) technologies on health care management and clinical decision-making [10,11,17-30].

Author, year	Country	Conditions	No of participants	Reported impact on clinical decision-making and health care management
Laverty et al [11] 2021	UK	Rheumatoid arthritis	20	Used PGHD to guide discussion during appointments No increase in appointment length reported
Lee et al [22] 2022	Malaysia	Chronic conditions	—^b^	Integration challenges identified
Richter et al [23] 2021	Germany	Rheumatoid arthritis	30	Provided a more comprehensive view of patient status Visualization style well-received by clinicians Data most useful during in-person visits
Rudin et al [19] 2019	USA	Asthma	26	PGHD useful for informing discussion Potential to reduce clinician workload Helped identify information patients may have forgotten to report Helped identify patients in need earlier
Sands et al [20] 2014	USA	Chronic conditions	—	Limited research currently available in this area Potential to drive innovation in care delivery
Skovlund et al [24] 2021	Denmark	Diabetes	12	PGHD supported person-centered care principles Data helped focus clinical discussions Use of PGHD increased patient engagement during appointments Training necessary for effective data use by clinicians
Solomon et al [21] 2022	USA	Rheumatoid arthritis	62	PGHD helped clinicians prepare for visits by capturing information that might otherwise be forgotten Use of PGHD may reduce unnecessary appointments and increase patient satisfaction
Trojan et al [25] 2021	Switzerland	Breast cancer	192	Use of PGHD positively impacted the quality of doctor visits
von Rohr et al [26] 2023	Germany	Axial spondyloarthritis	36	PGHD guided clinical decision-making PGHD enabled timely changes to treatment
A’Court et al [17] 2022	UK	Cardiovascular conditions	—	Self-monitoring devices contributed to clinical decision-making and management However, self-monitoring was not always perceived as helpful by users or clinicians
Arumalla et al [31] 2023	UK	Inflammatory arthritis	—	Use of PGHD was associated with a reduction in the number of required visits, although this finding was not statistically significant due to limited data
Cohen et al [10] 2016	USA	Asthma, cognitive decline, Crohn disease, premature infants	25 (5 studies)	PGHD increased clinicians’ understanding of patients’ illnesses, enabling better-informed visits and the identification of patients not meeting health care goals Use of PGHD was associated with a reduction in the number of visits Training is necessary for the effective use of PGHD Integration of PGHD with electronic patient record would improve accessibility Visualization and summarization of PGHD is helpful for clinicians to interpret data meaningfully Customization options supported HCPs'^c^ use of PGHD Patient engagement was essential to realize the full potential of PGHD
Austin et al [32] 2019	UK	Rheumatoid arthritis	40	Visualizations of PGHD supported person-centered consultations PGHD provided a more accurate reflection of patients’ experience of disease, enabling more personalized care Visualization of PGHD was perceived as time-saving for clinicians
Doyle et al [27] 2022	Ireland	Multimorbidity	120	PGHD provided clinicians with a better overview of the patient’s condition PGHD helped assess the effectiveness of patient self-management strategies Supported clinical decision-making during visits Emphasized the need to consider factors beyond PGHD in decision-making Reviewing PGHD before appointments was beneficial for clinicians Use of PGHD was associated with positive impacts on clinical outcomes Increased frequency of medication changes based on PGHD Unclear evidence on whether PGHD directly improved patient self-management
El Miedany et al [18] 2016	UK	Rheumatoid arthritis	211	PGHD enabled closer monitoring of disease activity and provided meaningful information for both patients and HCPs, supporting patient-centered care
Richter et al [28] 2021	Germany	Rheumatoid arthritis	60	PGHD facilitated patient-physician integration and improved communication PGHD made it easier to document and understand the course of the disease Provided a broader perspective on disease progression

a PGHD: patient-generated health data.

b Not available.

c HCPs: health care professionals.

### Characteristics of the Included Studies

The 16 included papers were published between 2014 and 2023 (Table 2). The majority were conducted in the United Kingdom (n=5, 31.25%) [11,17,18,31,32], followed by the United States (n=4, 25%) [10,19-21]. Most papers included were primary research papers (n=14, 87.5%) [10,11,17-19,21-28,32]. There were 2 literature reviews included in the analysis [20,31]. Within the original research, study sample sizes ranged from 12 to 211 participants. The most common disease featured in the papers was rheumatoid arthritis (n=6, 37.5%) [11,18,21,23,28,32], with the next most common defined as “chronic conditions” (n=2, 12.5%) [20,22]. Study designs varied, with a focus on early-stage research including 3 (18.75%) proof-of-concept studies [23,27,28], 2 (12.5%) feasibility studies [19,32], and 2 (12.5%) pilot studies, including both mixed methods formative [24] and prospective pilot studies [26]. This indicates a trend toward the preliminary testing of mHealth interventions prior to wider deployment. Other methods included interrupted time-series analysis [21], exploratory qualitative study [22], and mixed methods observational study [17].

### Potential Gap Between Study Aims and Focus

While many identified publications appeared relevant based on their titles and abstracts, a closer examination revealed a limited focus on the specific impact of PGHD on clinical decision-making or health care management. In several cases, such as in [29,30], the studies only tangentially addressed these themes, instead prioritizing broader topics such as the technical implementation of PGHD systems, data collection processes, or remote monitoring logistics.

This suggests that although interest in the role of PGHD within clinical settings is growing, empirical investigation into its direct influence on decision-making remains relatively underexplored. Some studies referenced potential benefits in this area but did not substantively examine them within their results or discussion sections and hence were not included in this review.

### PGHD mHealth Characteristics

Around 5 publications were part of other larger remote monitoring interventions, where PGHD was continuously collected outside of clinical settings and used to support long-term monitoring of conditions [18,19,21,23,24]. These interventions were often part of comprehensive programs aimed at improving patient outcomes by enabling regular self-monitoring and remote patient management.

A total of 9 papers incorporated PGHD mHealth tools alongside consultations [10,11,19,23-27,32]. In these cases, the data collected via mHealth apps were shared with health care providers during scheduled visits. Several studies described how this allowed clinicians to review trends or symptom reports in real time [11,17,21,23,26]; however, only 1 provided evidence on how this changed clinical decision-making [17]. Where described, the data were typically used to prompt discussion or support shared decision-making. Although some studies attempted to quantitatively assess the impact of PGHD on consultation time or the number of appointments needed [11,31], this was mostly inconclusive due to a lack of data. The subsequent search for newer papers identified authors quantifying the impact of remote PGHD, but they were excluded as they did not focus or explain how the approach interacted with health care behaviors or consultation style.

Around 7 publications reported integrating PGHD into electronic patient records (EPRs), allowing a seamless data flow between patient-reported information and clinical systems [11,18,19,21,23,24,32]. While some reported that clinicians perceived this integration as beneficial for enhancing the clinical visit [19,23], few papers evaluated whether this integration directly improved continuity of care. Descriptions generally focused on the technical integration and potential utility, rather than outcomes related to data use across settings or over time.

### Realizing Patient-Centered Care

A consistent feature of the literature was how PGHD collected through mHealth could act as a catalyst for person-centered care. This was based on the proposition that it can provide a holistic and accurate understanding of the person’s symptoms and health status. The data nurtured a more genuine partnership where professionals focused on the person’s needs and encouraged their input [10,18,23,24,27,32].

The presence of the PGHD altered consultation styles where health professionals invited individuals to offer the context about disease states, elicited new information together, and supported a focus on patient priorities [11,18,23,24,27]. There was also evidence that the PGHD could help align the clinician and patient agendas, forging a stronger relationship by validating an individual patient’s priorities and patients seeing value in what the professional wanted to measure, creating a collaborative common goal [10,22,24].

The literature highlighted the importance of supporting the health literacy of patients to enable them to engage in their own care and conversations with health professionals. Within this theme, it was noted that mHealth can empower patients to raise topics with health professionals, leading to changes in how the medical staff interacted, involved, and made treatment decisions with the patient. PGHD helped patients reflect on their disease, prepare for conversations they wanted to have, allowed parity in information access, and decreased power dynamics. In addition, it assisted patients by decreasing instances where they would forget what to raise, their priorities for the consultation, or how their quality of life had been in real time [21,24]. Correspondingly, health professionals altered their support by covering topics relevant to the individuals, personalizing thresholds for escalation, and tailoring care [18-21,24,26,27].

### Holistic Proactive Care

The literature showed that PGHD impacted how health care professionals made clinical decisions. The availability of data collected in between health care visits provided more complete clinical information leading to tailored management plans. The data mitigated issues with “snap shots” of symptoms, flawed clinic data (eg, white coat syndrome), and the temporal fluctuations in symptoms. The PGHD encouraged patients to explain contexts or triggers and attended to problems that may have been missed without the data, through early and focused support [10,11,22,26]. The patterns in the data, which could be viewed in advance, also allowed efficient use of time during consultations as perceived by the workforce. In addition, the use of mHealth technology also recorded prudent information that patients may be reluctant to share at consultations, which facilitated health care professionals offering holistic clinical support beyond typical disease−based outcomes (eg, sleep, erectile dysfunction) [19-22,27].

The access to PGHD data also modified health care decisions regarding service utilization, advice giving, and medication titration. Dashboard and alerts in real time led to health care professionals changing contact with patients, triggered both early hospitalization and deferring clinic visits, and augmented care, as patient goals could be monitored and supported [10,11,27,31]. There was also evidence of how PGHD can improve communication between professionals, particularly as it pertains to triggering triage or the need for a nonscheduled consultation [10,18,19,21,27].

### Data Functionality

Although the published papers did not demonstrate how the following aspects impact on health care decision-making, it was clear that changes to clinician behavior were contingent on elements related to the nature of the PGHD itself. It was noted in the papers that data accessibility will influence outcomes. The volume of data may be overwhelming, and relevant data need to be easily navigated and usable to allow positive impacts on health care decision-making. Likewise, summaries and visual representations are likely determinants of engagement with the data [10,22,24]. The integration of the data into workflows is key, as a lack of training, appropriate infrastructure, awareness, privacy issues, and information governance were highlighted as blockages and potential drains on workload resource [10,19,22,24].

### Reinforcing Medical Control

There were instances in the reviewed literature where PGHD were used to reinforce a biomedical paradigm. There were occasions where professionals used data to verify verbal accounts, challenge patients, and highlight discrepancies on what patients reported [11,21]. In other publications, professionals’ actions showcased paternalism by using data to highlight patients who were not meeting treatment goals and trying to persuade or nudge them to adhere to treatment plans [21,27]. Likewise, despite the potential for shared decision-making, in 1 paper, it was the clinicians who decided what data would be discussed, when the data would be discussed, and the data’s relevance to the treatment [11]. The PGHD could be used to keep power with the clinicians. This was demonstrated where clinicians downplayed PGHD as subjective when convincing the patient about treatment options, which did not align with patients' views and advocating the objectivity of PGHD when it supported their clinical decisions [11].

Finally, it was noted that health care professionals were hesitant to rely on PGHD, questioned its validity, and were concerned that patients may confabulate the findings, showcasing an enduring reverence for medically led data. There was a yearning for additional rigor, standards for practice, and reliance on medical procedures to inform decisions due to a lack of trust in the data and nonstandardization of data collection via mHealth [10,21,22].

## Discussion

### Principal Findings

The findings from this review highlight both the promise and challenges of utilizing PGHD collected via mHealth to support clinical decision-making and health care delivery, underscoring a dynamic interplay between patient empowerment, clinical decision-making, and systemic implementation barriers. While PGHD have demonstrated their capacity to support personalized care and enrich clinical decision-making, integration is hindered by persistent concerns around data usability, professional skepticism, and structural limitations within health care systems.

Given the heterogeneity of the included studies, the findings can be conceptually organized into 3 overlapping roles of PGHD: informational (supporting clinical knowledge and risk assessment), operational (shaping workflow, triage, or pathway redesign), and relational (influencing patient-provider communication and autonomy). This categorization was introduced retrospectively as a lens to help interpret the evidence, rather than being applied during data extraction or synthesis. It provides a way to understand how PGHD may influence both clinical decision-making and broader health care management while retaining the exploratory nature of the scoping review.

PGHD has been positioned to enhance patient autonomy and shared decision-making, offering clinicians a more comprehensive view of patient experiences beyond episodic consultations [10,29]. The ability of mHealth PGHD to facilitate deeper engagement in care planning was confirmed in this review where data-informed consultations led to more tailored treatment strategies and improved alignment with patient needs [10,18,23,24,27,32]. However, despite this potential, there remains a gap between the intended function of mHealth PGHD and its actual use in practice. Other literature has substantiated these findings, noting similar challenges in integrating PGHD, with Omoloja and Vindavalli [33] noting that challenges include the inability to incorporate PGHD into clinical workflows.

Findings from this review suggest that, in some cases, mHealth PGHD has been used to reinforce traditional clinical authority rather than shift toward a more patient-led approach. While the intent is often to empower patients, professionals may use PGHD to validate clinical perspectives or steer patients toward predetermined treatment pathways [11,21,27]. Concerns over the authentic sharing of power and misuse of data have been confirmed elsewhere. For instance, it has been shown that mHealth tools can inadvertently structure consultations around patient noncompliance, potentially silencing patient voices and undermining empowerment [34]. At the same time, the findings indicate that PGHD can also facilitate the redistribution of decision-making and collaborative care, depending on how data are interpreted, discussed, and integrated into clinical workflows.

Despite the potential, the successful integration of mHealth PGHD into clinical workflows is impeded by practical and structural barriers. Technological constraints, particularly difficulties in integrating with EPR systems, remain a persistent issue. Many studies highlighted professional concerns around data accuracy, completeness, and relevance, contributing to a reluctance to incorporate mHealth PGHD into routine decision-making [10,21,22]. Furthermore, the volume of mHealth PGHD presents usability challenges; without appropriate filtering and summarization tools, professionals may struggle to extract actionable insights, leading to disengagement with mHealth platforms [10,19,22,24]. These findings about the challenges in integrating PGHD into clinical workflows are echoed elsewhere in a scoping review [35], which highlighted that PGHD integration is still in its infancy and that few studies detail successful incorporation into EPRs.

Beyond technological constraints, implementation issues also stem from organizational and policy-level challenges. A lack of standardized frameworks for PGHD governance, coupled with limited training and institutional support, has resulted in inconsistencies in adoption [10,24]. Without structured integration strategies, mHealth PGHD risks become an additional burden rather than a facilitator of efficiency, reinforcing professional hesitancy and limiting scalability [10,19,22,24]. The negative impact of poorly curated data systems on staff burnout, safety, staff retention, and hesitancy to engage has also been discussed in the wider literature [36] where the volume and complexity of data increased clinician burnout. This knowledge underscores the need for better organizational strategies to support sustainable PGHD use. Having an integrated strategy is one of the World Health Organization’s digital guiding principles, noting that without this, the initiatives may result in information fragmentation and the poor delivery of services [37].

The review showed that mHealth PGHD is a mechanism for optimizing health care efficiency, offering real-time insights that can inform service utilization, triage decisions, and proactive intervention strategies [38]. Some studies highlighted its role in reducing unnecessary clinic visits, improving medication titration decisions, and enhancing remote monitoring capabilities, contributing to more sustainable health care models [10,11,27,31]. However, while these findings are promising, evidence on the long-term impact of PGHD remains limited. Other authors [35] also note that PGHD integrations are often in the pilot phase, with few studies measuring prudent outcomes, indicating that development and testing are still at a preliminary stage.

Many of the included studies focused on feasibility rather than effectiveness, highlighting a gap in empirical validation of mHealth PGHD’s impact on clinical and economic outcomes [19,23,24,26-28,32]. Without robust longitudinal data, it remains unclear whether PGHD can drive meaningful improvements in resource utilization and health care system efficiency beyond pilot implementations.

Although this narrative scoping review provides a broad overview of the literature, there are several limitations that can be noted. First, there is an obvious publication bias in the literature where most of the included studies were from high-income, Western countries, which potentially limits the generalizability of the findings. Also, it should be noted that the heterogeneity in the study designs could make direct comparisons challenging. Despite these limitations, this review provides a comprehensive synthesis of the existing evidence on mHealth PGHD’s impact on clinical decision-making and service design, highlighting key areas for future research.

Future research should focus on the development of standardized PGHD integration models, ensuring that data are presented in a clinically meaningful and interpretable format. Additionally, further exploration of how PGHD reshapes patient-health care provider dynamics is essential to safeguarding against unintended consequences, such as the reinforcement of traditional clinical authority rather than true patient empowerment.

Longitudinal studies assessing PGHD’s impact on clinical workflows, decision-making, and health care resource utilization will be crucial in determining its viability as a core component of health care delivery.

### Conclusion

This review demonstrated that mHealth PGHD has the potential to enhance patient-centered care, improve clinical workflows, and optimize resource utilization, yet challenges remain in its implementation due to technological barriers and professional acceptance. While mHealth PGHD may support proactive care and reduce unnecessary clinic visits, mHealth PGHD’s role in shared decision-making appears mixed, sometimes reinforcing existing clinical hierarchies. To realize the full potential of mHealth PGHD, future research should focus on standardized integration models, effective data presentation, and the evolving dynamics between patients and providers. Establishing clear policy frameworks and addressing these gaps is crucial for establishing PGHD as a new facet of health care delivery. Despite limitations such as study heterogeneity, this review offers valuable insights to inform future implementation and policy development.

## Supplementary material

10.2196/77359 Multimedia Appendix 1 Full search strategy.

10.2196/77359 Multimedia Appendix 2 Full data extraction sheet.

10.2196/77359 Checklist 1 PRISMA scoping review checklist.

## Abbreviations

EPR: electronic patient record
LTHC: long-term health condition
MeSH: Medical Subject Headings
PGHD: patient-generated health data
PRISMA-ScR: Preferred Reporting Items for Systematic Reviews and Meta-Analyses extension for Scoping Reviews
