# Supplementary material for: The Impact of Patient-Generated Health Data From Mobile Health Technologies on Health Care Management and Clinical Decision-Making: Narrative Scoping Review
Source: J Med Internet Res. 2025 Dec 19;27:e77359. doi: 10.2196/77359 (PMC12716635; doi:10.2196/77359)
Supplement: Multimedia Appendix 1 [file jmir-v27-e77359-s001.docx]

**Evidence Search Results**

| **Search question:**  *What is the impact of patient reported outcome measures (PROMs) or patient-generated health data via mobile apps on digital transformation of long-term condition management?* |
| --- |
| **Results**  Relevant natural language and controlled vocabulary terms were selected and combined. Final result sets were de-duplicated and reviewed for relevance by the searcher, irrelevant results being discarded.  As discussed, the resulting 234 articles have been downloaded as a RIS file |
| **Contents**  [Results as RIS file 2](#_Toc152943289)  [Search history & disclaimer 3](#_Toc152943290) |
| Links to guidance and other resources from relevant organisations found through a general internet search are provided at the start. Further results from a search of the clinical databases are grouped by type and listed with abstracts below the summary in publication date order, with links to full text where available.  **For full-text availability, please click on the links below the article citation. If an article is not available, please complete the request form that populates via the article link, and we will obtain the article for you**. |

**Evidence search results**

# Results as RIS file

**______________________________________________________**

# **Search history & disclaimer**

**Approach**

Relevant natural language and controlled vocabulary terms were selected and combined. Final result sets were de-duplicated and reviewed for relevance by the searcher, irrelevant results being discarded.

**Search strategy**

| **Resources searched:** Embase, Medline, Knowledge & Library Hub, British Nursing Index, Proquest Health Research Premium Collection |
| --- |
| **Limits:** No time limit, English-language only, not children or adolescents |

**Sample search strategy - Medline & Embase via Ovid**

Embase <1974 to 2023 December 06>

Ovid MEDLINE(R) ALL <1946 to December 06, 2023>

1 exp Chronic Disease/ or exp Heart Diseases/ or Diabetes Mellitus/ or exp Joint Diseases/ or exp Inflammatory Bowel Diseases/ or exp Respiratory Tract Diseases/ or exp Connective Tissue Diseases/ 10830224

2 ("long-term condition*" or co-morbidit* or multi-morbidit*).mp. [mp=ti, ab, hw, tn, ot, dm, mf, dv, kf, fx, dq, bt, nm, ox, px, rx, ui, sy, ux, mx] 101377

3 (chronic* adj (condition* or disease* or ill*)).mp. 713106

4 (copd or chronic obstructive pulmonary disorder or asthma or cystic fibrosis or bronchiectasis or pulmonary fibrosis).mp. [mp=ti, ab, hw, tn, ot, dm, mf, dv, kf, fx, dq, bt, nm, ox, px, rx, ui, sy, ux, mx] 933849

5 (diabet* or heart failure or hypertension or heart disease* or cardiomyopath*).mp. [mp=ti, ab, hw, tn, ot, dm, mf, dv, kf, fx, dq, bt, nm, ox, px, rx, ui, sy, ux, mx] 4819131

6 (rheumat* or arthritis or spondylitis).mp. [mp=ti, ab, hw, tn, ot, dm, mf, dv, kf, fx, dq, bt, nm, ox, px, rx, ui, sy, ux, mx] 924651

7 1 or 2 or 3 or 4 or 5 or 6 13481979

8 exp Ambulatory Care/ or exp Outpatient Clinics, Hospital/ or exp Ambulatory Care Facilities/ 252824

9 (ambulatory care or outpatient* or (clinic* adj2 visit*)).mp. [mp=ti, ab, hw, tn, ot, dm, mf, dv, kf, fx, dq, bt, nm, ox, px, rx, ui, sy, ux, mx] 788921

10 8 or 9 829826

11 exp Patient Reported Outcome Measures/ or exp Routinely Collected Health Data/ or exp Patient Generated Health Data/ 405900

12 ("patient reported outcome measure*" or proms or eproms or "e-proms" or experience measure* or symptom tracker* or self-monitor*).mp. [mp=ti, ab, hw, tn, ot, dm, mf, dv, kf, fx, dq, bt, nm, ox, px, rx, ui, sy, ux, mx] 82762

13 ((patient-generated or patient-reported or self-generated or self-reported) adj4 (data or outcome* or measure*)).mp. [mp=ti, ab, hw, tn, ot, dm, mf, dv, kf, fx, dq, bt, nm, ox, px, rx, ui, sy, ux, mx] 189016

14 ("patient-initiated" or "clinician-initiated").mp. 2928

15 11 or 12 or 13 or 14 562518

16 exp Mobile Applications/ 38522

17 ((app or mobile* or phone or smart phone* or smartphone*) adj3 (data or collect* or captur* or monitor* or record*)).mp. [mp=ti, ab, hw, tn, ot, dm, mf, dv, kf, fx, dq, bt, nm, ox, px, rx, ui, sy, ux, mx] 23284

18 ("mobile e-health" or "m-health" or mhealth or (remote adj monitor*) or wearable*).mp. [mp=ti, ab, hw, tn, ot, dm, mf, dv, kf, fx, dq, bt, nm, ox, px, rx, ui, sy, ux, mx] 92053

19 16 or 17 or 18 139231

20 7 and 10 and 15 and 19 393

21 (appointment* or visit* or follow-up*).af. 4949876

22 20 and 21 263

23 22 use medall 63

24 exp chronic disease/ or exp heart disease/ or exp diabetes mellitus/ or exp arthropathy/ or exp inflammatory bowel disease/ or exp obstructive airway disease/ or exp connective tissue disease/ 8115254

25 ("long-term condition*" or co-morbidit* or multi-morbidit*).mp. 101377

26 (chronic* adj (condition* or disease* or ill*)).mp. 713106

27 (copd or chronic obstructive pulmonary disorder or asthma or cystic fibrosis or bronchiectasis or pulmonary fibrosis).mp. 933849

28 (diabet* or heart failure or hypertension or heart disease* or cardiomyopath*).mp. 4819131

29 (rheumat* or arthritis or spondylitis).mp. 924651

30 24 or 25 or 26 or 27 or 28 or 29 10461004

31 exp outpatient/ or exp emergency outpatient clinic/ or exp outpatient department/ or exp outpatient care/ 349586

32 exp ambulatory care/ 110958

33 (ambulatory care or outpatient* or (clinic* adj2 visit*)).mp. 788921

34 31 or 32 or 33 810630

35 exp patient-reported outcome/ or exp routinely collected health data/ 72431

36 ("patient reported outcome measure*" or proms or eproms or "e-proms" or experience measure* or symptom tracker* or self-monitor*).mp. 82762

37 ((patient-generated or patient-reported or self-generated or self-reported) adj4 (data or outcome* or measure*)).mp. 189016

38 ("patient-initiated" or "clinician-initiated").mp. 2928

39 36 or 37 or 38 230109

40 exp mobile application/ 38522

41 ((app or mobile* or phone or smart phone* or smartphone*) adj3 (data or collect* or captur* or monitor* or record*)).mp. 23284

42 ("mobile e-health" or "m-health" or mhealth or (remote adj monitor*) or wearable*).mp. 92053

43 40 or 41 or 42 139231

44 30 and 34 and 39 and 43 269

45 (appointment* or visit* or follow-up*).af. 4949876

46 44 and 45 184

47 44 use oemezd 172

48 23 or 47 235

49 remove duplicates from 48 198

**Disclaimer**

**Any queries on specific drug regimens or dosages should be passed to the Medicines Information Service on ext. 55304.**

Searching the literature retrieved the information provided. We recommend checking the relevance and critically appraising the information contained within when applying to your own decisions as we cannot accept any responsibility for actions taken based on this information. Every effort has been made to ensure that the information supplied is accurate, current and complete, however for various reasons it may not represent the entire body of information available.

We would also draw your attention to the inherent publication bias in the evidence base towards predominantly white, northern hemisphere countries. This means the evidence found might not be relevant to all patient populations.

**Acknowledgement**

If you have found this service valuable please tell your colleagues! Please also consider acknowledging the Library & Knowledge Service’s role in any subsequent report, publication or presentation. Please acknowledge this work in any paper or presentation as:

Evidence Search: Research title. Librarian name. xx month 202x. Torquay, UK: South Devon Health Library & Knowledge Service
